# CXCR1/2 antagonism with CXCL8/Interleukin-8 analogue CXCL8_(3–72)_K11R/G31P restricts lung cancer growth by inhibiting tumor cell proliferation and suppressing angiogenesis

**DOI:** 10.18632/oncotarget.4066

**Published:** 2015-06-10

**Authors:** Muhammad Noman Khan, Bing Wang, Jing Wei, Yingqiu Zhang, Qiang Li, Xuelin Luan, Jya-Wei Cheng, John R. Gordon, Fang Li, Han Liu

**Affiliations:** ^1^ Institute of Cancer Stem Cell, Dalian Medical University, Dalian, China; ^2^ Department of Immunology, Dalian Medical University, Dalian, China; ^3^ Jilin Medical College, Jilin, China; ^4^ Institute of Biotechnology, Department of Life Science, National Tsing Hua University, Hsinchu, Taiwan; ^5^ The Division of Respirology, Critical Care and Sleep Medicine, Royal University Hospital, University of Saskatchewan, Saskatoon, Canada

**Keywords:** lung cancer, G31P, CXCR antagonist, ELR-CXC chemokine

## Abstract

CXCR1 and CXCR2 together with cognate chemokines are significantly upregulated in a number of cancers, where they act as key regulators of tumor cell proliferation, metastasis, and angiogenesis. We have previously reported a mutant protein of CXCL8/Interleukin-8, CXCL8_(3–72)_K11R/G31P (G31P), which can act as a selective antagonist towards CXCR1/2 with therapeutic efficacy in both inflammatory diseases and malignancies. In this study, we investigated the effect of this ELR-CXC chemokine antagonist G31P on human non-small cell lung cancer cells and lung tumor progression in an orthotopic xenograft model. We report increased mRNA levels of CXCR1 and CXCR2 in human lung cancer tissues compared to normal counterparts. Expression levels of CXCR1/2 cognate ligands was determined by ELISA. CXCR1/2 receptor antagonism via G31P leads to decreased H460 and A549 cell proliferation and migration in a dose-dependent manner. G31P also enhanced apoptosis in lung cancer cells as determined by elevated levels of cleaved PARP, Caspase-8, and Bax, together with a reduced expression of the anti-apoptotic protein Bcl-2. In an *in vivo* orthotopic xenograft mouse model of human lung cancer, G31P treatment suppressed tumor growth, metastasis, and angiogenesis. At the molecular level, G31P treatment was correlated with decreased expression of VEGF and NFкB-p65, in addition to reduced phosphorylation of ERK1/2 and AKT. Our results suggest that G31P blockage of CXCR1 and CXCR2 can inhibit human lung cancer cell growth and metastasis, which offers potential therapeutic opportunities.

## INTRODUCTION

Lung cancer is a leading cause of death in the world. The mortality rate due to lung cancer has increased over the past 3 decades, surpassing liver cancer as the leading cause of death due to malignant tumors in China [1, 2]. Globally, lung cancers are the leading cancer of both males and females [3]. The majority of patients diagnosed with lung cancer has incurable advanced disease with few therapeutics options. Inspiringly, recent targeted therapy approaches against Epidermal Growth Factor Receptor (EGFR) and Anaplastic Lymphoma Receptor Tyrosine Kinase (ALK) have shown promising results and been approved for clinical use. Nevertheless, this group only accounts for a small proportion of lung cancer, and the remaining majority still lacks a druggable target till now.

It is widely accepted that inflammation is closely implicated in cancer development and progression. Chemokines are secreted proteins that regulate cell behavior via their cognate G-protein coupled receptors (GPCRs), with four families of chemokines coordinating tissue inflammation by recruiting and activating leukocytes [4]. Constitutive expression of proinflammatory chemokines is already described in many cancer types, which supports tumor growth through direct stimulation of cancer cells or establishment of supportive tumor stroma, in addition to facilitate cancer cell invasion [5, 6]. Lung cancer malignancies are often linked to abnormal expression or activation of an array of growth factors, cytokines, ELR-CXC chemokines, and their receptors, which cumulatively activate signaling pathways that foster tumor growth [7, 8].

The CXCR1 and CXCR2 GPCRs are important therapeutic targets in many solid tumors, including lung, breast, prostate, and ovarian cancers, hepatocellular carcinoma and melanoma [9–12]. Evidences suggest that CXCR2 may participate directly in tumor progression [13]. For example, melanoma tumor progression and lung metastasis are significantly inhibited in CXCR2-deficient mice compared to wild type mice [14]. Furthermore, it has also been shown in a murine model that depletion of CXCR2 inhibits tumor growth and angiogenesis of lung cancer [15]. The seven sibling ELR-CXC chemokines (i.e. CXCL1–3 & 5–8) can bind and activate either CXCR1 or CXCR2 receptor [16]. Previous studies have confirmed that these chemokines can drive tumor growth in the absence of other inflammatory changes, but also foster the development of tumor resistance to chemotherapy [16]. CXCL8, for example, is an effective and potent angiogenic factor [17]. Many non-small cell lung cancer cell lines produce CXCL8, the expression of which is related to angiogenesis, metastasis, and poor survival rates in many studies. Thus, targeting the mitogenic and angiogenic activities of these chemokines will help to control tumor invasion and metastasis of human lung cancers [18–20]. These data suggest that CXCR1/2 antagonists that bind with high affinity to these receptors and block downstream signaling pathways can be used for greater therapeutic responsiveness and efficacy in non-small cell lung cancers.

We have previously reported a CXCL8/Interleukin-8 analogue, CXCL8_(3–72)_K11R/G31P (or briefly G31P), which contains Arg and Pro substitutions for Lys11 and Gly31 respectively of human CXCL8_(3–72)_. G31P is a selective CXCR1 and CXCR2 antagonist, which has shown high efficacy in the treatment of multiple inflammatory diseases [21–23]. Moreover, we also explored the potential effects of G31P during tumorigenesis. G31P treatment inhibits human prostate cancer growth and metastasis in a mouse model by decreasing tumor cell proliferation and angiogenesis [24]. More recently, our further study also proved effectiveness of G31P in promoting apoptosis of hepatocellular carcinoma cells [25]. On the basis of above information, we hypothesized that inhibition of CXCR1 and CXCR2 by G31P might attenuate tumorigenesis of lung cancer. We evaluated the potency of G31P as a therapeutic approach in non-small cell lung cancer, investigating its impact on tumor cell proliferation, metastasis, and angiogenesis. We also elucidate the molecular mechanism by which CXCR1/2 antagonism with G31P inhibits downstream signaling during tumor proliferation and metastasis. Our results suggest that use of G31P with or without other chemotherapeutic drugs would improve therapeutic benefits in the treatment of lung cancer.

## RESULTS

### CXCR1/2 expression in human lung cancer cell lines and tissues

**Figure 1:**
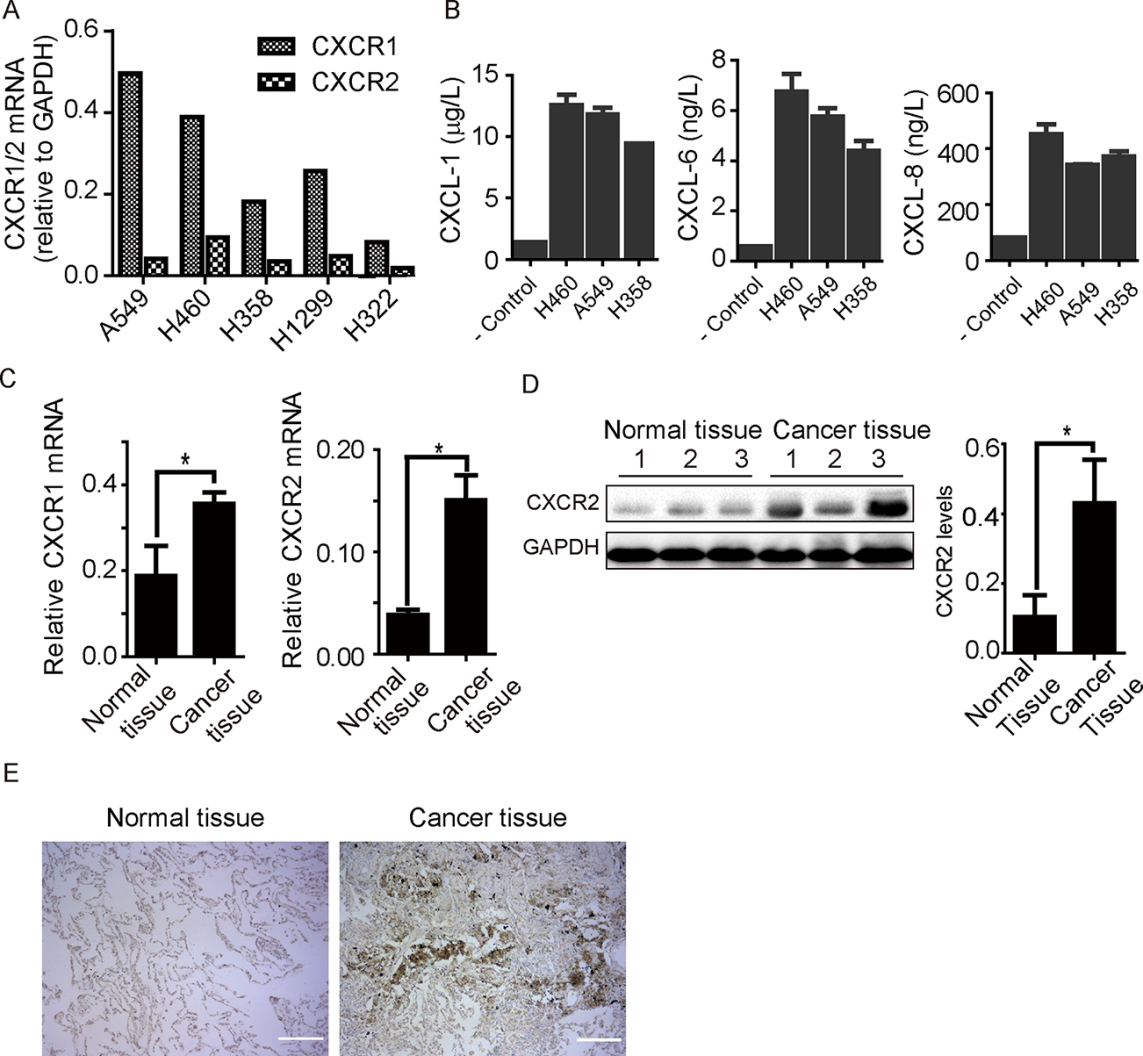
Expression of CXCR1 and CXCR2 receptors in human lung cancer tissue and cell lines **A.** CXCR1 and CXCR2 mRNA was detected in a panel of non-small cell lung cancer cell lines as indicated. All five cell lines show CXCR1 and CXCR2 mRNA expression. **B.** CXCL1, 6, and 8 chemokines were detected from conditioned media of H460, A549, and H358 through ELISA. Data are summarized from 3 independent experiments; error bars represent SEM (standard error of the mean). **C.** CXCR1 and CXCR2 mRNA expression was quantified through PCR in human lung cancer tissue comparing to adjacent non-cancerous tissue from same patients (*n* = 8). CXCR1 and CXCR2 mRNA was expressed more in cancer tissue than non-cancerous counterpart. Results represent mean ± SEM (*, *p* < 0.05). **D.** protein expression and quantification histogram represent the presence of CXCR2 receptor in non-cancerous and cancer tissues of human samples, (*, *p* < 0.05). **E.** immunohistochemistry results of CXCR2 expression in normal and cancer tissues of human lung samples. Scale bar = 200 μm.

As non-small cell lung cancer (NSCLC) forms the majority of lung cancer, we focus our efforts on NSCLC in this study. We examined mRNA levels of CXCR1 and CXCR2 in a panel of non-small cell lung cancer cell lines including A549, H460, H358, H1299, and H322. All cancer cells express both CXCR1 and CXCR2, which is consistent with previous data reported by Zhu et al. [18]. In comparison, H358 and H322 appear to contain lower amounts of CXCR1 and CXCR2 mRNA than A549, H460, and H1299, while A549 and H460 harbor highest quantities of mRNA for CXCR1 and CXCR2 respectively (Figure 1A). Additionally, we assessed secretion of CXCL1, CXCL6, and CXCL8, chemokines for CXCR1/2, in conditioned medium from NSCLC cells using enzyme-linked immunosorbent assay (ELISA). As expected, secretion of CXCL1, CXCL6, and CXCL8 is evident for H460, A549, and H358, with highest amounts from H460 and A549 cells (Figure 1B). We also compared mRNA levels of CXCR1 and CXCR2 in lung cancer and normal tissues from same patient (a group of 8). Both CXCR1 and CXCR2 predominately expressed in cancer tissues. As shown in Figure 1C, CXCR1 mRNA was present in normal tissues but at a lower level than in cancer tissues. CXCR2 mRNA was almost deficient in normal tissues but noticeably present in cancer tissues. We then inspected CXCR2 protein expression in cancer and non-cancerous tissues by immunoblotting and immunohistochemistry. CXCR2 predominately expressed in cancer tissue than normal tissue of human lungs (Figure 1D and 1E). Taken together, these findings provide further evidence that CXCR1/2 and their ligands are upregulated in lung cancer. Since H460 and A549 express the most CXCR1 or CXCR2, these two cell lines were chosen for following studies.

### ELR-CXC chemokine antagonism inhibits NSCLC cell proliferation

**Figure 2:**
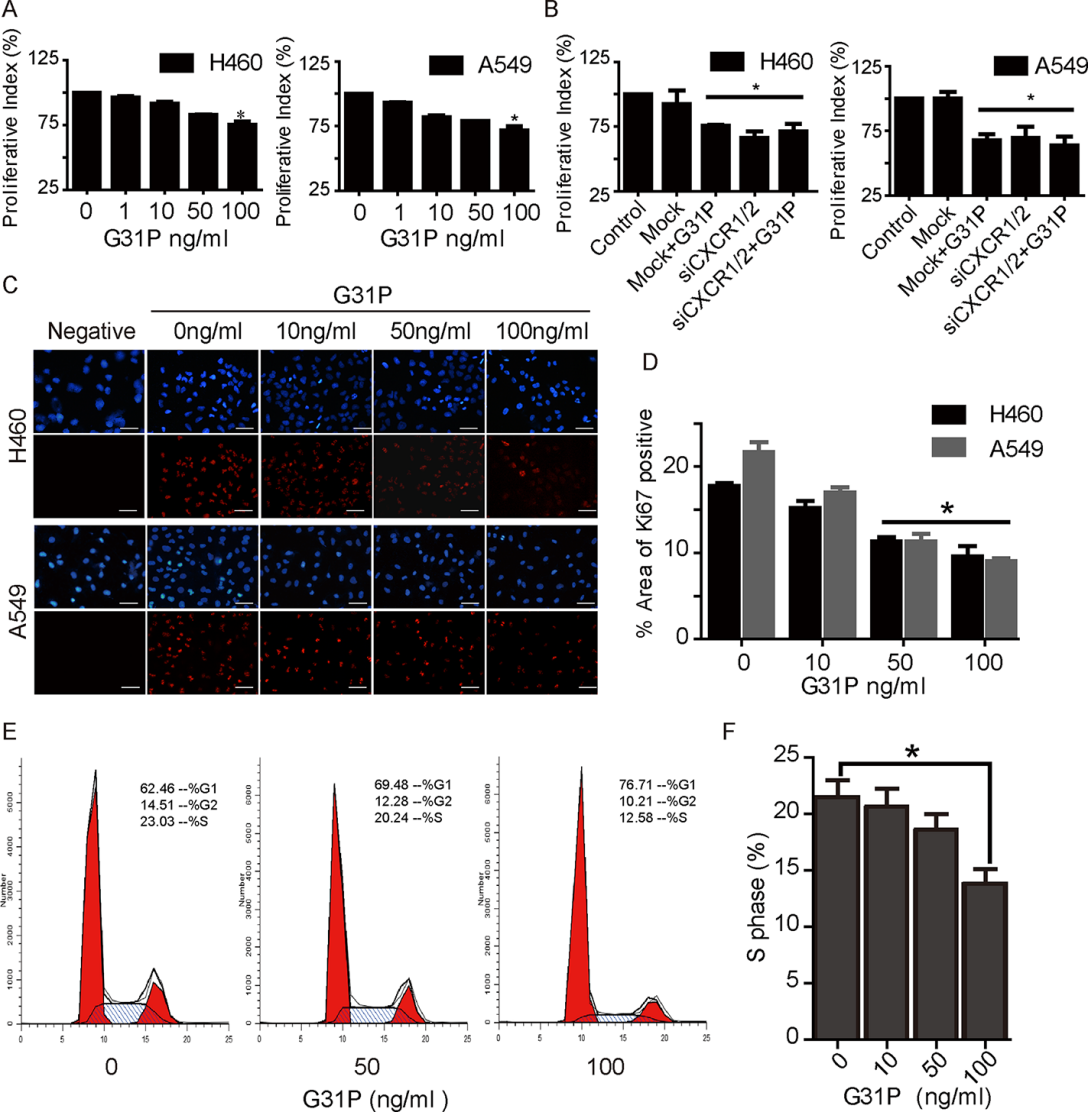
CXCR1/2 antagonism by G31P inhibits NSCLC cell proliferation **A.** cells were treated with G31P (at concentrations of 0, 1, 10, 50, and 100 ng/ml) for 48 h. Cell proliferation was measured by CCK8 assay at 450 nm. G31P at 100 ng/ml showed significant inhibition of growth, (*, *p* < 0.05). **B.** cells treated with CXCR1/2 siRNA or control reagents were assessed for proliferation with or without G31P. G31P and siCXCR1/2 showed similar reduction but with no additive effect (*, *p* < 0.05). **C.** validation of G31P effect on H460 and A549 cell proliferation by Ki-67 nuclear stain through immunofluorescence. Ki-67 protein expression (red fluorescence) was detected significantly lower in G31P treated cells when compared with control for both cell lines, scale bar = 100 μm. **D.** graph represents percentages of area with positive Ki-67 stain (mean ± SEM) from three independent experiments (*, *p* < 0.05). **E.** cell cycle analysis of G31P-treated H460 cells shows reduction of cells in S and G2/M phases. **F.** graph represents percentages of cells in S phase after G31P treatment. All error bars represent standard error of the mean (SEM), and * indicates *p* < 0.05. All data were summarized from at least 3 independent experiments.

It has been reported that the expression levels of some ELR-CXC chemokines is prognostic of patient outcomes in multiple cancers [26]. Given our observation that non-small cell lines express augmented levels of CXCR1 and CXCR2, we next assessed whether CXCR1/2 antagonism with CXCL8_(3–72)_K11R/G31P (hereafter G31P) could affect the proliferation of these cells. We have previously reported on the development and activities of G31P in multiple models, including some cancers [21–25]. We assessed the effect of increasing concentrations of G31P on H460 and A549 cell proliferation *in vitro*, using CCK-8 assay. Both cell lines were sensitive to G31P, which reduced their proliferation in a dose-dependent manner (Figure 2A). When used at 100 ng/ml, G31P inhibited H460 and A549 cell growth significantly (27% and 30% respectively) compared to untreated cells. In order to figure out whether such effect was mediated by CXCR1/2, we carried out knockdown experiments with siRNAs targeting CXCR1/2 and repeated cell proliferation assays. CXCR1/2 knockdown led to comparable reduction in cell proliferation with G31P treatment (100 ng/ml), and combined treatment of both did not show additive anti-proliferative effect (Figure 2B). These results suggest that G31P exerts its anti-proliferative effect through CXCR1/2 receptors. Further, we examined impact of G31P on expression of the proliferative marker Ki-67 in these cells by immunofluorescence. G31P treatment reduced nuclear expression of Ki-67 in both populations of cells (Figure 2C and 2D). Finally, we looked at cell cycle distribution of H460 cells by flow cytometry following G31P treatment. Results showed that G31P (100 ng/ml) significantly reduced proportion of cells in S phase of cell cycle (Figure 2E and 2F).

### G31P suppresses cell migration

**Figure 3:**
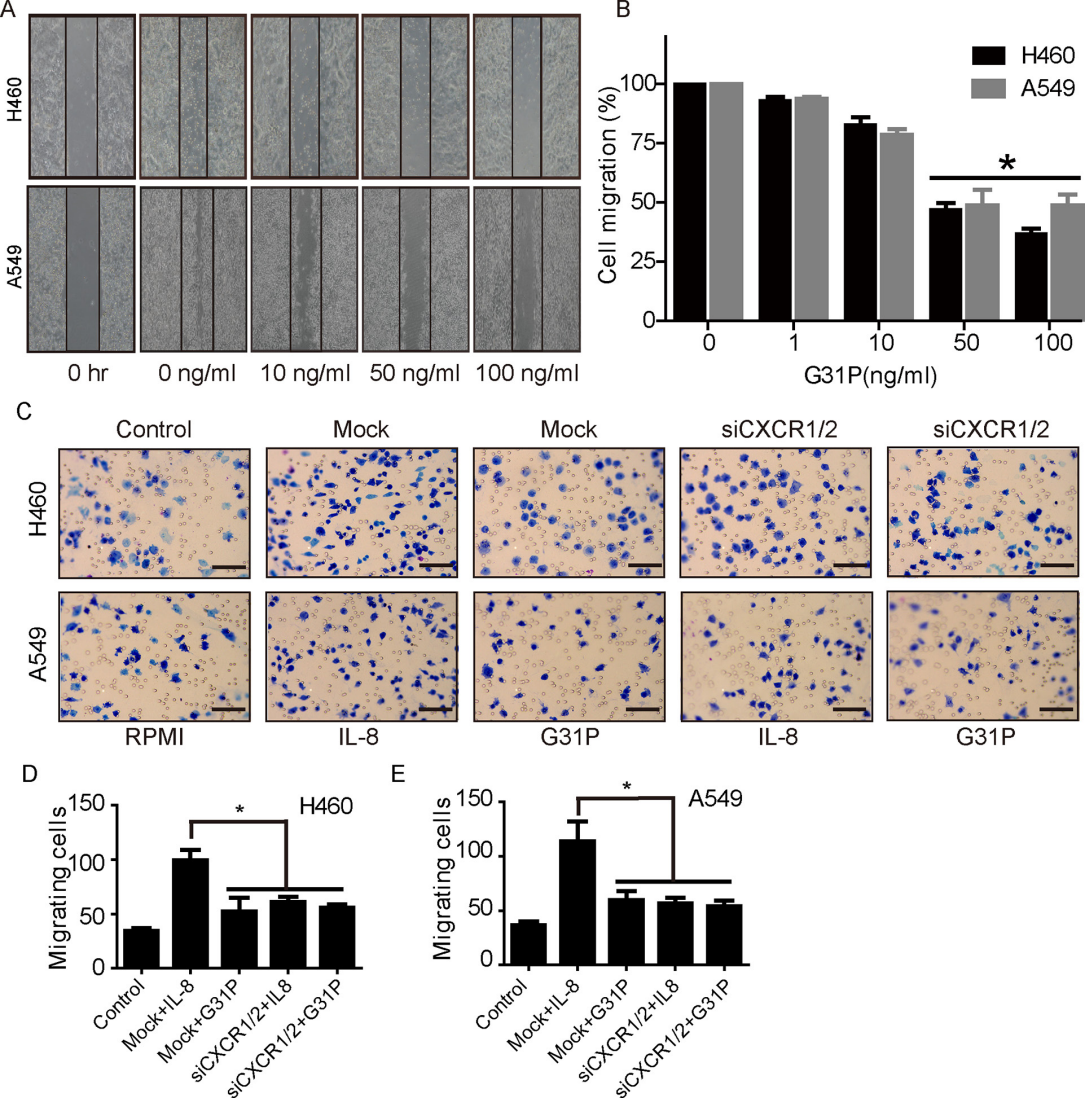
G31P restricts migratory and chemokinetic capacities of H460 and A549 cells **A.** migration of H460 and A549 cells was assessed by wound healing assay and migration rate (%) was calculated and demonstrated in **B.** The data depicted represent the relative migration rate of H460 and A549 as quantified by measuring the distance covered by cells during 24 h. **C** and **D.** impact of G31P on chemotaxis of H460 and A549 cells, as determined using modified Boyden chamber microchemotaxis assays. CXCR1/2 and mock siRNA transfected cells were loaded into upper chamber and CXCL8/IL-8 (20 ng/ml) and G31P (100 ng/ml) were filled into lower chambers. Representative images were shown in C. Histograms in D show that G31P and siCXCR1/2 treatment inhibit lung cancer cell migration. Graph represents mean ± SEM from three independent experiments. Scale bar = 100 μm, and (*, *p* < 0.05).

As another means of evaluating the impact of ELR-CXC chemokine antagonism on lung cancer cell vitality, we examined the effect of G31P on the migratory abilities of both H460 and A549 cells, using ‘wound healing’ and chemokinesis assays. We found that cells treated with increasing concentrations of G31P showed impaired wound closure when compared with untreated group that nearly closed the gap. We observed that G31P treatment with 50 and 100 ng/ml significantly reduced the migrating capability of lung cancer cells (to 46.89% and 39.48% for H460 while 51.37% and 48.76% for A549 respectively, Figure 3A and 3B). In addition, we assessed whether ELR-CXC chemokine antagonism could affect chemokinetic movement of tumor cells in modified Boyden chamber assays. The upper chamber of each well was loaded with cells and lower chambers with growth media either as is or together with G31P (100 ng/ml) and IL-8 (20 ng/ml). After 2 h, we enumerated the cells that had migrated through polycarbonate membrane into the lower wells. As expected, both populations displayed substantial chemokinetic activity, which was further enhanced by IL-8. Addition of G31P reduced cell migration significantly, which was phenocopied by CXCR1/2 knockdown, while G31P treated siCXCR1/2 cells also exhibited resembling defect. Represented photomicrographs of Giemsa stained cells are shown in Figure 3C and quantification in Figure 3D. Our findings demonstrate that G31P has significant inhibitory effects on migration of non-small cell lung cancer H460 and A549 cells.

### ELR-CXC chemokine antagonism induces apoptosis

**Figure 4:**
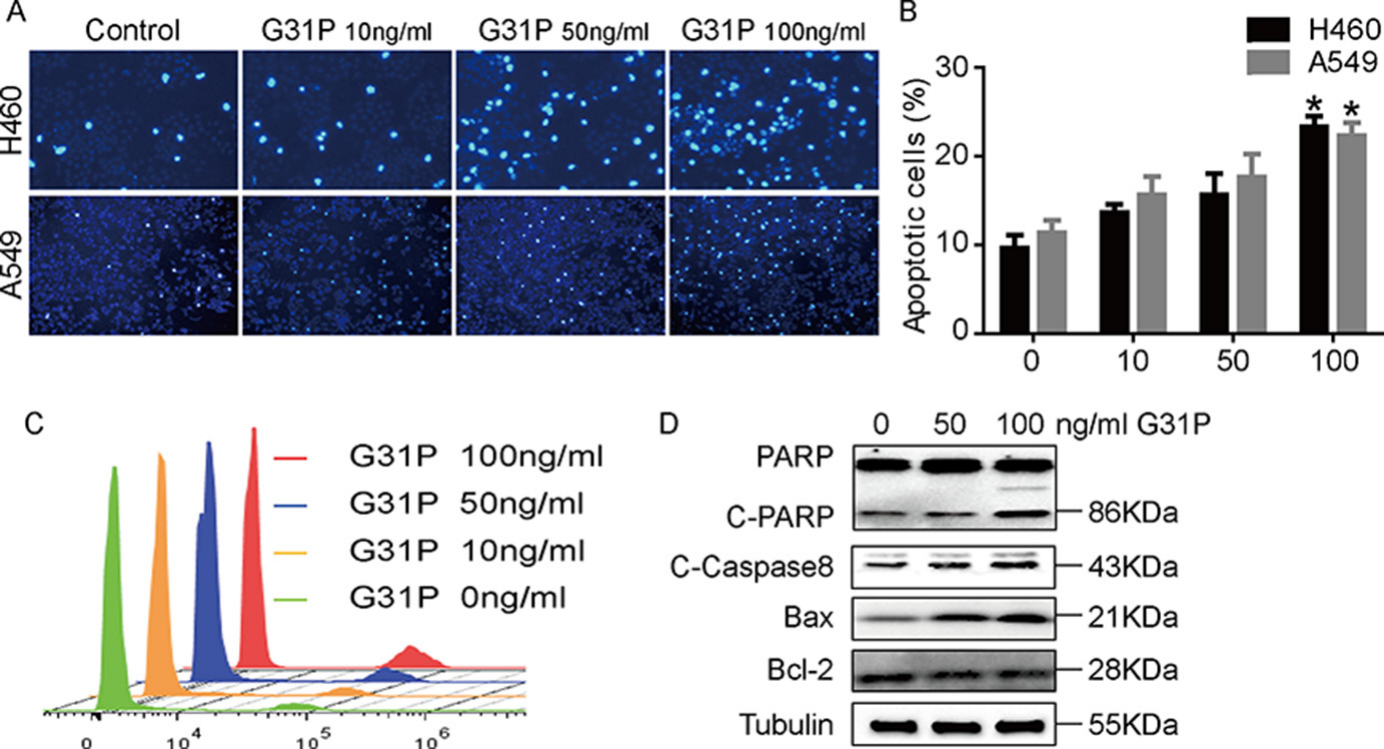
CXCR1 and CXCR2 antagonism induces apoptosis in NSCLC cells **A.** H460 and A549 cells were treated with the indicated concentrations of G31P and assessed for uptake of dye Hoechst 33342. **B.** quantification data from A show percentages of apoptotic cells. Data were summarized from 3 independent experiments and error bars represent SEM. **C.** flow cytometric analysis of H460 cells treated with indicated concentrations of G31P by staining with Hoechst 33342. Results show a peak of intact cells on the left of each track and a second peak of apoptotic cells. **D.** impact of G31P on expression of the indicated apoptosis-associated proteins in H460 cells. G31P treatment augmented expression of cleaved PARP and Caspase-8, as well as Bax, but modestly reduced expression of the anti-apoptotic protein Bcl-2, α-Tubulin was used as loading control.

We next assessed the apoptotic index in H460 and A549 cells and the impact of ELR-CXC chemokine antagonism on this process, as determined by staining with Hoechst 33342. This reagent stains the condensed chromatin of apoptotic cells more brightly than the DNA of non-apoptotic cells. As shown in Figure 4A, G31P treatment increased the staining intensity of both H460 and A549 cells. It induced 20 to 30% of H460 cells to become apoptotic, whereas 18 to 25% of A549 were apoptotic after 48 h of G31P treatment (Figure 4B). We confirmed this by flow cytometry where elevating concentrations of G31P increased the amounts of apoptotic cells (Figure 4C). Further, we performed immunoblotting experiments to inspect expression of a range of apoptosis related proteins, including PARP, Caspase-8, Bcl-2, and Bax. We observed elevation of cleaved PARP and Caspase-8 as well as Bax but a decrease in the anti-apoptotic Bcl-2 following G31P addition, which collectively indicated a more apoptotic characteristics compared to untreated group (Figure 4D). These results suggest that G31P induces apoptosis by regulating expression of these proteins.

### G31P inhibits localized lung cancer growth and metastasis in nude mice

**Figure 5:**
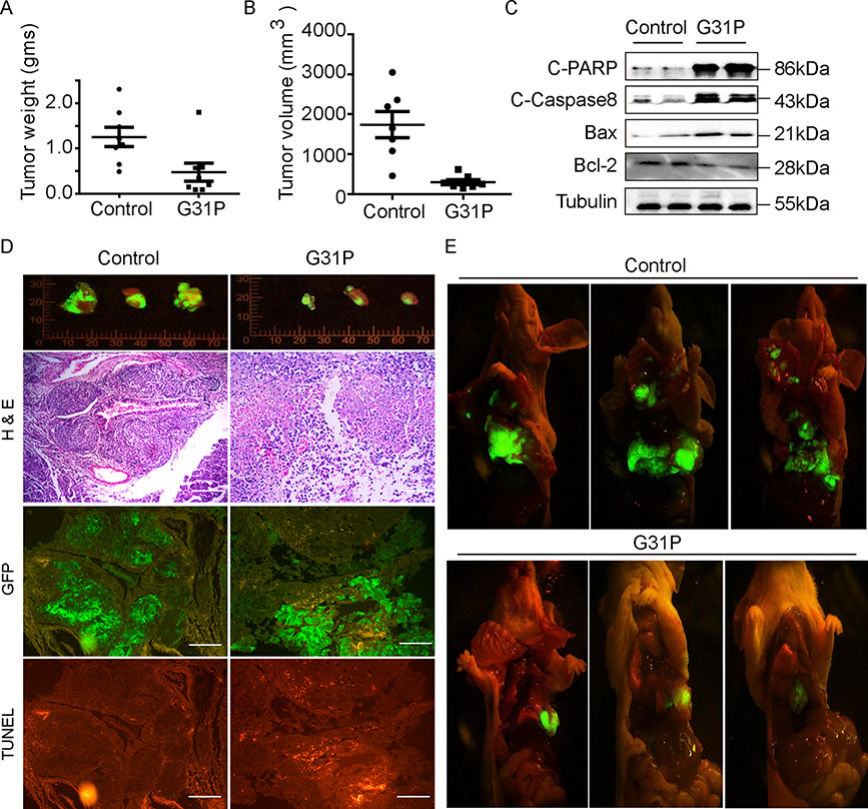
G31P inhibits H460 xenograft growth and metastasis in mouse model **A and B.** weights and volume of the xenografts from each mouse were assessed and plotted respectively. Tumors from the G31P-treated mice were significantly smaller than those from the saline-treated mice (*p* < 0.01). **C.** impact of G31P on expression of the indicated apoptosis-associated proteins in xenograft tumors. G31P treatment increased expression of cleaved PARP, Caspase-8, and Bax, while mildly decreased amounts of Bcl-2. α-Tubulin was used as loading control. **D.** gross fluorescent pictures were taken of GFP-expressing H460 cell tumors resected from saline- (control) and G31P-treated mice, 3 from each are shown as examples. G31P effects on apoptosis and necrosis of tumor were confirmed by serial sections stained with H & E and TUNEL. GFP fluorescent micrograph was also taken to differentiate live and necrotic part of tissue. H & E staining confirmed more nucleated cells/necrosis ratio in control group than G31P group, whereas TUNEL staining of tumor serial section micrographs exhibits more apoptosis in G31P treated tumor. Scale bar = 100 μm. **E.** representative fluorescent images (open chest and abdomen cavities) of GFP-expressing H460 tumors in saline (control) and G31P treated mice (*n* = 8 in each group). The tumor volume and extent of metastasis were substantially decreased in the G31P-treated mice. Error bars represent standard error of the mean.

To evaluate the effect of G31P on lung cancer growth and metastasis *in vivo*, we established an orthotopic lung cancer model in nude mice with GFP expressing H460 cells. Mice were treated with G31P (500 μg/kg) one week post tumor implantation for four weeks. Fluorescent images of GFP expressing tumor in mice demonstrated that G31P treatment significantly reduced lung cancer formation and growth. Moreover, tumor weight was reduced 64.25% ± 6.37% and tumor volume was reduced 75.56% ± 9.30% relative to control treated (Figure 5A and 5B). Histologically, tumors in the G31P treatment group included large areas of necrotic cells, with viable neoplastic cells comprising only a small portion of the tissue within G31P-treated tumors. In contrast, essentially the entire tumor mass within vehicle-treated mice comprised viable tumor cells (Figure 5D). TUNEL staining of tumor sections showed that G31P treated tumors contained more apoptotic cells (TUNEL positive cells) and smaller number of live cells (GFP positive cells) than control group (Figure 5D). We also assessed the impact of ELR-CXC chemokine antagonism on tumor metastasis in this model. Control group mice displayed metastasis throughout their thoracic cavities, including to muscles and lymph nodes, as compared to G31P-treated mice, wherein the tumors largely remained localized to their primary implantation sites, with negligible metastasis being discernible (Figure 5E). Consistent with abovementioned data from cancer cell lines, immunoblotting analyses of apoptotic proteins including cleaved PARP, cleaved Caspase-8 and Bax from G31P treated xenografts showed increased intensity than control samples, with concurrent decreased expression of the anti-apoptotic protein Bcl-2 (Figure 5C).

### G31P suppresses tumor angiogenesis in mice model

**Figure 6:**
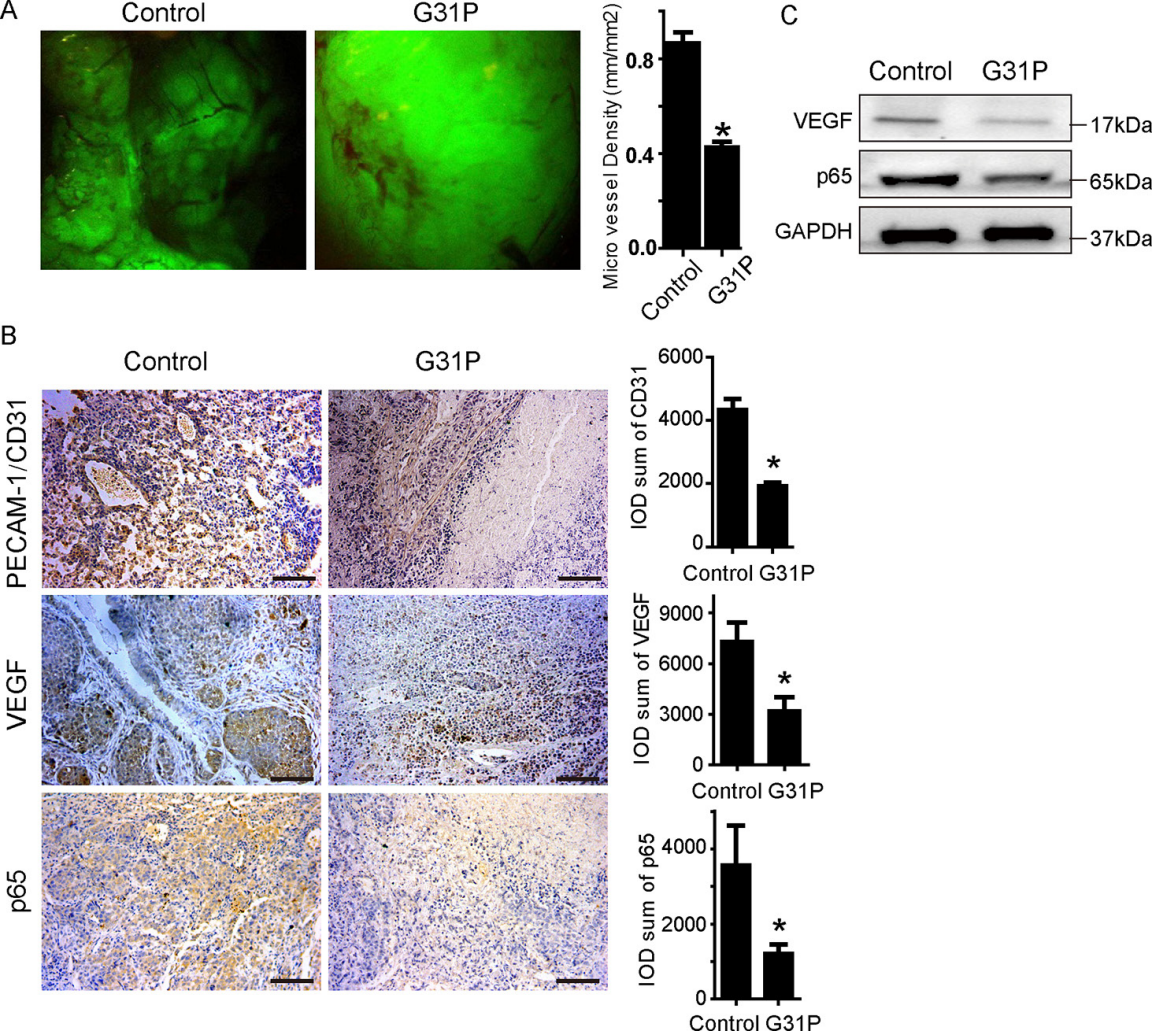
G31P suppresses angiogenesis in xenograft tumor **A.** gross photographs of H460 tumors growing in nude mice. Right side chart shows quantification data in which microvessel length and tumor areas were measured to calculate microvessel densities (MVD) using the formula: density = microvessel length/xenograft area. The graph represents the summarized MVD data with mean ± SEM from 6 mice in each group. **B.** tissue sections from control and G31P-treated xenografts were analyzed by immunohistochemistry for PECAM-1/CD31, VEGF and NFкB-p65 expression, scale bar = 100 μm. Right side charts show quantification data calculated as integral optical density (IOD) of PECAM-1/CD31, VEGF and NFкB-p65. Reduced expression was evident with G31P. **C.** immunoblottings for VEGF and NFкB-p65 in resected tumors from control and G31P-treated mice show reduced VEGF and p65 levels. GAPDH was used as loading control. All error bars represent standard error of the mean, and * indicates *p* < 0.05.

An important facet of tumor progression is the achievement of adequate vascular development to support the growing tumor mass. As such, we studied the impact of G31P treatment on a number of angiogenesis markers in our tumor-bearing mice. We captured fluorescent images of the tumors using a dissecting microscope and calculated the microvessel densities of each tumor using Image-Pro software. The mean vascular density (MVD) of the tumors in the G31P-treated group was 0.45 ± 0.028 mm/mm^2^, while that in the control group was 0.930 ± 0.04 mm/mm^2^ (Figure 6A). We then examined the expression of PECAM-1/CD31, VEGF and NFкB-p65 proteins within the tumors using immunohistochemistry. CD31 and VEGF are closely implicated in angiogenesis, while NFкB-p65 is an inflammatory modulator and angiogenic factor [27]. Immunohistochemical staining of tumor sections for CD31, VEGF and NFкB-p65 was quantified by IOD (integrated optical density), which showed that G31P significantly reduced expression of these important angiogenesis related proteins (Figure 6B). Immunoblotting analyses of VEGF and NFкB-p65 showed that ELR-CXC chemokine antagonism reduced expression of both proteins in xenografts relative to vehicle-treated tumors (Figure 6C). These results confirm that G31P treatment leads to reduced expression of proteins involved in the formation of new blood vessels and thus displays potent anti-angiogenic activity.

### G31P attenuates activation of MAPK and AKT

**Figure 7:**
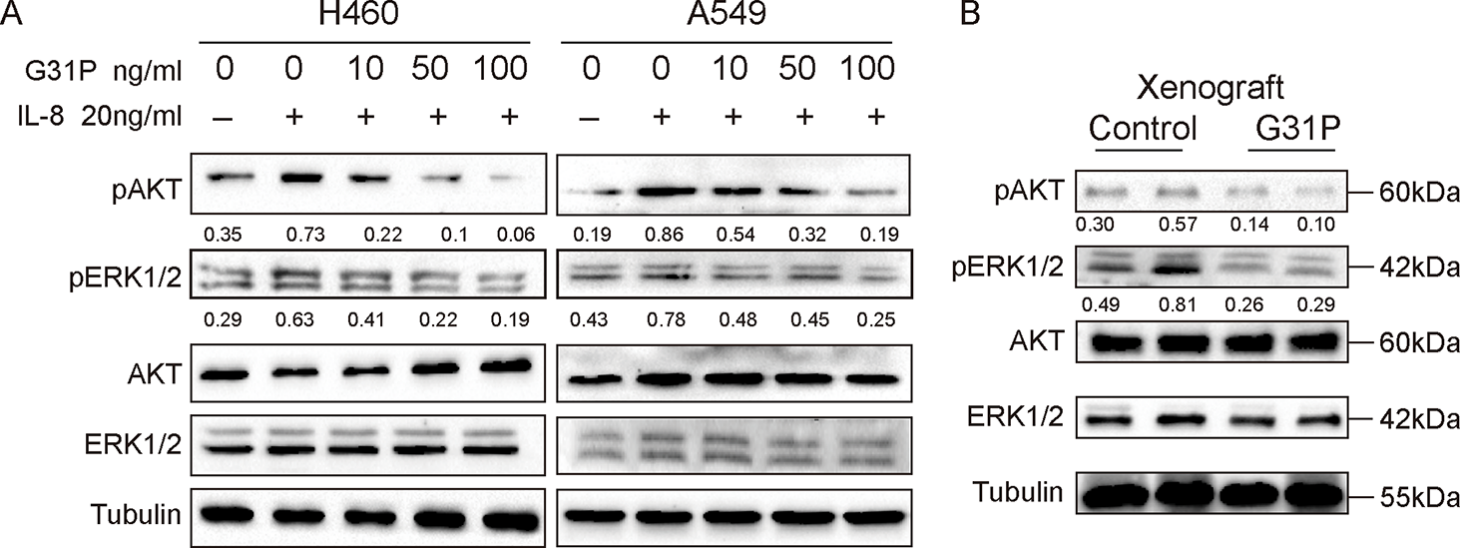
G31P treatment decreases the levels of pAKT and pERK1/2 in lung cancer cells and xenografts **A.** H460 and A549 cells were treated with G31P (concentrations of 0, 10, 50 and 100 ng/ml) for one h before stimulated with IL-8 (20 ng/ml) for 30 minutes. Results showed that G31P treatment with increasing concentrations suppressed the IL-8 induced upregulation of pERK1/2 and pAKT in both cell lines. **B.** similarly in xenografts decreased phosphorylation of ERK1/2 and AKT is evident. α-Tubulin was used as loading control, against which normalized intensity was shown for pAKT and pERK1/2.

It is well-recognized that the expression levels of ELR-CXC chemokines correlates with activation of various signal transduction pathways for a number of tumors. Hence we assessed whether G31P treatment would affect expression of the activated forms of MAPK (ERK1/2) and AKT in xenografts or among non-small cell lung cancer cell lines. H460 and A549 cells were pretreated with G31P at different concentrations and then stimulated with IL-8 (20 ng/ml) for 30 minutes. Proteins were isolated to determine the levels of phosphorylated forms of ERK1/2 and AKT. In both H460 and A549 cells, G31P treated cells showed reduced phosphorylation of AKT and moderately decreased pERK1/2 levels, although total AKT and ERK1/2 were unaffected (Figure 7A). H460 displays more reduced activation of AKT and ERK1/2 than in A549 cells. We also examined phosphorylation of these proteins in xenograft tissues from our tumor bearing mice. Samples from mice treated with vehicle (control group) showed significantly more phosphorylation of ERK1/2 and AKT than those from G31P treated mice (Figure 7B). Therefore, our *in vivo* and *in vitro* data are consistent, which together suggest that CXCR1/2 antagonism by G31P inhibits the activation of MAPK and AKT signaling pathways that play pivotal roles in lung cancer progression.

## DISCUSSION

Lung cancer is a leading cause of cancer-related death in industrialized countries, largely because of the advanced stage at which it is often diagnosed and of its poor response to the presently available treatments [3]. As such, new, targeted treatment approaches are required. CXCL8_(3–72)_K11R/G31P (G31P) is a low molecular weight ELR-CXC chemokine inhibitor that has a higher binding affinity for CXCR1 and CXCR2 than CXCL8 or any of its sibling ELR-CXC chemokines [28]. This study focused on the impact of CXCR1 and CXCR2 antagonism by G31P on proliferation, migration, survival and growth of non-small cell carcinoma cells and tumors *in vitro* and *in vivo*, the latter as determined in a xenograft mouse model. Our results clearly indicate that G31P has inhibitory effects on lung cancer growth, metastasis, and angiogenesis, at least in part through its effects on the MAPK and AKT signaling pathways.

The contribution of ELR-CXC chemokines such as CXCL8/IL-8 and its receptors CXCR1/2 to cancer progression, including non-small cell lung cancer, has been well-documented [18–19]. Clinical studies have confirmed that increased expression of CXCL8 is linked with tumor development, suggesting that suppression of ELR-CXC chemokines may be an important therapeutic approach for aggressive and metastatic tumors. Expression of CXCL8 and its receptors correlates with angiogenesis, tumor progression, and poor survival in non-small cell lung cancer [20]. Indeed, using neutralizing antibodies to IL-8, Arenberg and colleagues reported reduced tumor size and decreased spontaneous metastasis in human non-small cell lung cancer mice model [29]. Many non-small cell lung cancer cell lines, including H460, A549, and H358 were shown to produce CXCL8 and other ELR-CXC chemokines, while small cell lung cancers produced low or insignificant levels of CXCL8 [30, 31]. In the present study, we report that a number of human lung cancer cell lines, as well as freshly-resected tumor tissues from cancer patients, express both CXCL8 receptors CXCR1/2, which are consistent with previous studies. It has been suggested that angiogenesis in human NSCLC is mediated more by CXCR2 than CXCR1, possibly due to the fact that CXCR2 is a receptor for a more diverse array of ELR-CXC chemokines than CXCR1 [13–15]. In support of this notion, we did observe that non-small cell lung cancer cell lines secreted several prominent ELR-CXC chemokines, CXCL1/growth-related oncogene alpha (GROα), CXCL6/granulocyte chemotactic protein 2 (GCP-2), and CXCL8/IL-8.

Several small molecule inhibitors that target ELR-CXC chemokine receptor signaling have been developed to suppress inflammatory disease and tumor growth [32]. In contrast, G31P is an engineered analogue of CXCL8 (i.e. CXCL8_(3–72)_K11R/G31P) that effectively antagonizes all seven ELR-CXC chemokines, but also desensitizes signaling via heterologous G protein-coupled receptors on CXCR1- and/or CXCR2-expressing cells. G31P has been shown to have important anti-inflammatory activities in many forms of lung injury [21–23], and we have reported on its protective effects as an anti-cancer drug in hepatocellular carcinoma and prostate cancers [24–25]. Herein we further evaluate its efficacy in non-small cell lung cancer.

Upregulation of ELR-CXC chemokine signaling promotes tumor cell proliferation and survival, mainly through modulating cell cycle progression and apoptosis [16]. Silencing of CXCR2 controls cellular apoptosis by affecting the expression of pro-apoptotic factors, leading to cell cycle arrest in the G0-G1 and G2-M phases [15]. Our *in vitro* proliferative assay demonstrates that G31P has dose-dependent inhibitory effects on lung cancer cell proliferation, also supported by data from Ki-67 nuclear staining. In agreement, treatment with G31P led to H460 accumulation in the sub G1 phase of the cell cycle. Additionally, G31P suppressed migratory capacities of non-small cell lung cancer cells, as indicated by results from wound healing and modified Boyden chamber experiments. These defects appeared to be associated with CXCR1/2 expression as suggested by our siRNA mediated knockdown experiments. G31P also stimulated apoptosis, as revealed by increases in the expression of Bax, cleavage of PARP and Caspase-8, together with increase in Hoechst 33342 staining. These *in vitro* effects were validated *in vivo* as well by our demonstration that G31P treatment of tumor-bearing mice led to dramatic reduction in tumor volume, weight, and metastasis, with augmented tumor cell apoptosis.

Heterotopic lung cancer models have been criticized as not representative of lung cancers, in as much as the primary tumor is anatomically distant from the lung. To overcome this we used an orthotopic model in which we implanted the primary tumor, with metastasis driving its movement into nearby regions or lymph nodes. During cancer growth, vascularization is a key factor to support the development of tumors, wherein CXCL8 induces endothelial growth through CXCR1 and CXCR2 signaling [33–36]. Our *in vivo* results showed that G31P-treated mice had substantially smaller primary tumors, with little discernible tumor metastasis. Regarding impact on angiogenesis, we observed decreases in VEGF and NFкB-p65 expression in tumor tissues from G31P-treated mice, with corresponding reduction in microvessel density of these tumors. We also observed increased apoptosis in G31P treated tumor using TUNEL staining, supported by immunoblotting analyses of apoptotic proteins such as PARP, Caspase-8, BAX, and Bcl-2. This pro-apoptotic effect of G31P is likely further enhanced *in vivo* through its inhibition on tumor tissue vascularization.

ELR-CXC chemokines stimulate a wide array of downstream signaling molecules through binding to CXCR1 and CXCR2, among which activation of MAPK and AKT is closely implicated in cancer development and progression. Upregulation of phosphorylated forms of ERK1/2 and AKT has been detected in many cancers, promoting tumor proliferation, invasiveness, and metastasis, while inhibiting apoptosis. We observed that ELR-CXC chemokine antagonism with G31P was associated with attenuated signaling through each of these within non-small cell carcinoma cell lines as well as within intact tumors growing in nude mice. Of note, we observed stronger reduction of the activation of ERK1/2 *in vivo* compared to in cell lines, which probably reflects the additional effect of G31P on tumor microenvironment that offers input to the Ras-MAPK cascade. These results indicate the molecular mechanism by which G31P suppresses tumor growth and induces apoptosis.

In conclusion, this study provides significant evidence that CXCR1 and CXCR2 antagonism with G31P inhibits lung cancer growth and metastasis, through directly restraining cancer cell proliferation and migration as well as stimulating apoptosis, but also indirectly modulating tumor microenvironment by suppressing angiogenesis. These events correlate with effects on phosphorylation of ERK1/2 and AKT, together with expression levels of VEGF and NFкB-p65 at the molecular level. Our findings collectively suggest that G31P, via targeting CXCR1/2 signaling, can be considered as an important factor in therapeutic strategies designed for the clinical management of non-small cell lung cancer.

## MATERIALS AND METHODS

All experiments were performed in accordance with the 1996 National Institutes of Health Guide for the Care and use of Laboratory Animals, and the experimental procedures were approved by the Local Committee of Animal Use and Protection.

### Antibodies and other reagents

The following antibodies were used: rabbit anti-CXCR2 (Abcam, USA), mouse anti-Ki67 (BD Biosciences, USA), rabbit anti-Akt, rabbit anti-phospho-Akt (Ser473), rabbit anti-ERK1/2, mouse anti-phospho-ERK1/2 and PARP (Cell Signaling Technologies, USA), mouse anti-GAPDH, rabbit anti-Caspase-8, rabbit anti-Bax and Bcl-2 (ProteinTech, USA), and rabbit anti-VEGF and NFкB (Santa Cruz), mouse anti-α-tubulin (Sigma Aldrich). G31P is prepared as described previously [21, 22].

### Cell culture and human samples

NSCLC lung cancer cell lines H460, A549, H1299, H358, and H322 were cultured in complete RPMI-1640 medium containing 10% fetal bovine serum and 1% penicillin/streptomycin (Hyclone) at 37°C in a humidified incubator containing 5% CO_2_. Human lung cancer and nearby noncancerous biopsies (*n* = 8) were obtained from the 1^st^ Affiliated Hospital of Dalian Medical University, Liaoning, China. Human samples were collected in accordance with the guidelines approved by the Institutional Committee.

### siRNA transfection

Transfection was performed using Lipofectamine 2000 (Life technologies) according to manufacturer's instructions. siRNA sequences used are as follow: CXCR1, 5′-GGAGUUCUUGGCACGUCAUCGUGUU-3′ (forward) and 5′-AACACGAUGACGUGCCAAGAACUCC-3′ (reverse); CXCR2, 5′-ACCGAGAUUCUGGGCAU CCUUCACA-3′ (forward) and 5′- UGUGAAGG AUGCCCAGAAUCUCGGU-3′ (reverse). In brief, 1 × 10^5^ H460 and A549 cells were plated in RPMI medium with 10% FBS. Next day, cells were treated with siRNA targeting CXCR1/2 or control reagents. Cells were incubated at 37°C for 48 or 72 h and knockdown efficiency was assessed by RT-PCR and immunoblotting.

### Cell proliferation assay

Cell proliferation assay was performed using cell counting kit-8 (CCK-8) (Dojindo, Kumamoto, Japan). H460 and A549 cells were plated in 96 well plates at 5000 cells/well and cultured for 12 h in 10% FBS containing RPMI growth medium. Cells were treated with G31P at concentrations of 1, 10, 50 and 100 ng/ml. Equal volume of growth media without G31P was added in control wells. Cells were maintained at 37°C in a humidified incubator. After 48 h, G31P containing media was replaced from wells with 110 μl of RPMI containing 10 μl CCK-8 solution. After an additional incubation for 4 h, absorbance of each well was determine at 450 nm and cell proliferation rate was calculated. Proliferation rates of siRNA transfected H460 and A549 cells treated with or without G31P were assessed in the same way after 48 h of transfection.

### Ki-67 stain

Cells were cultured on glass coverslips. After 24 h of G31P treatment, cells were fixed in 4% paraformaldehyde for 20 minutes before treated with 0.2% Triton-PBS for 10 minutes. Cells were incubated in 2% BSA for 30 minutes and then with anti-Ki-67 (BD Biosciences) for 30 minutes at room temperature. After 3 times PBS washes PE-conjugated secondary antibody was added for 30 minutes, followed by three times PBS washes, and coverslips were mounted on slide with 50 μl mowiol supplemented with DAPI. Images were captured using inverted fluorescent microscope (BX-51, TR32000 Olympus, Japan) and Ki-67 expression was calculated.

### Wound healing assay

Wound healing assay was performed as described previously [24]. In brief, cells were cultured in 12 well plates to reach at least 80% confluence. Using a 200 μl pipette tip, a wound was created in confluent monolayer of cells and wells were rinsed twice with PBS. Next, cells were incubated for 24 h in complete medium containing G31P (at concentrations of 10, 50, and 100 ng/ml) at 37°C. Cell migration was evaluated using inverted microscope (Leica, Germany). Wound healing rate was quantified as distance cells migrated across the injury line during 24 h of incubation.

### Modified Boyden chamber chemokinesis assay

H460 and A549 cell migration was assessed using modified Boyden chamber (Neuroprobe, Gaithersburg, MD) microchemokinesis assays with 8 μm-pore sized, fibronectin-coated polycarbonate membranes. CXCR1/2 siRNA and control treated cells in RPMI 1640 were loaded into upper chamber and G31P (100 ng/ml) and IL-8 (20 ng/ml) in RPMI medium were filled in lower chamber. In control group the lower chambers were filled with RPMI 1640. After 2 h at 37°C, cells that had migrated to the lower surface of filter membrane were fixed in methanol and stained with Giemsa solution. The mean number of cells contained within microscopic fields was determined by direct counting using a microscope (Leica, Germany). Cells were counted from at least 3 wells from same group in single experiment.

### Apoptosis assay

H460 and A549 cells cultured in RPMI-1640 media were seeded in 6 well plate. After 12 h starvation, G31P was added to final concentrations of 0, 10, 50, and 100 ng/ml. After 48 h, cells were washed with PBS and stained with Hoechst 33342 (5 μg/ml). Incubation time was 30 minutes at 4°C. Images were taken from 5 randomly chosen fields under fluorescent microscope (BX-51, TR32000 Olympus, Japan). More than 100 cells per field were counted and graph was plotted with ratios of live and apoptotic cells. In flow cytometry, H460 cells were seeded in 6 cm plate. After G31P treatment (up to 100 ng/ml) for 24 h, cells were harvested and washed with PBS at 4°C and adjusted to cell density of 1 × 10^6^ cells/ml in PBS. Cells were stained with Hoechst 33342 (5 μg/ml) for 5 minutes. After staining, cells were immediately analyzed with an automatic flow cytometer (Accuri C6, BD Biosciences). Data were analyzed with FlowJo 7.6.1.

### Cell cycle analysis

To analyze cell cycle distribution, 1 × 10^6^ cells were harvested and washed with PBS, then resuspended in 1 ml PBS. The cells were fixed in 9 ml ice cold 75% ethanol at 4°C overnight and then washed with PBS and resuspended in propidium iodide (20 μg/ml in PBS with 0.1% Triton-X 100, Sigma Aldrich) and RNaseI (100 μg/ml, Sigma Aldrich). Samples were incubated at 37°C in water bath for 15 minutes and analyzed by flow cytometer (Accuri C6, BD Biosciences).

### CXCR1/2 mRNA expression

Total RNA was extracted from human tissue and non-small cell lung cancer cell lines including H460, A549, H1299, H358 and H322 using Trizol reagent according to instructions (TaKaRa Bio, Dalian). cDNA was reverse transcribed using Prime Script RT reagent Kit (TaKaRa Bio, Dalian). QPCR reaction was performed following manufacturer's instructions (TaKaRa Bio) with a thermal cycler (BioRad, USA). Relative mRNA expression of each gene was normalized to GAPDH levels. Primers used are as follow, CXCR1 (NM_000634.2) 5′-GAGCCCCGAATCTGACATTA-3′ (forward, 1466–1485) and 5′-GCAGACACTGCAACACACCT-3′ (reverse, 1646–1665); CXCR2 (NM_001557.3) 5′- ATTCTGGGCATC CTTCACAG-3′ (forward, 1319–1338) and 5′-TGCAC TTAGGCAGGAGGTCT-3′ (reverse, 1501–1520); GAPDH (NM_002046.5) 5′-CCATCACTGCCACCCAGA AGAC-3′ (forward, 727–748) and 5′-ATGACCTTGCC CACAGCCTTG-3′ (reverse, 830–850).

### Enzyme-linked immunosorbent assay

The expression of CXCL1, CXCL6, and CXCL8 by different lung cancer cell lines was assessed using ELISA kits (Senbeijia Nanjing Biotechnology Co. Ltd.). After 24 h starvation, supernatant was collected and immediately processed for ELISA as instructed by the manufacturer.

### GFP expressing cell line

To visualize orthotopic lung cancer cells in nude mice, H460 cells were transfected with GFP expressing vector. In brief, pEGFP plasmid was obtained from TaKaRa biotechnology (Dalian, China). In transfection, H460 cells were treated with 1:1 mixture of precipitated retroviral PT67 cells (GFP expression packaging cells) supernatant supplemented with RPMI-1640 with 10% FBS. After 72 h medium was changed to selective medium containing G418. Stable GFP expressing clones were picked and expanded using conventional culture method

### Orthotopic lung cancer model in nude mice

Athymic female nude mice (4–6 weeks old Balb/c background) were maintained in laminar airflow cabinets under specific pathogen-free conditions, and provided free access to irradiated pellet food and sterilized water. For the xenograft model, GFP-expressing H460 cells were inoculated subcutaneously in the right flank of three nude mice (5 × 10^6^ cells/0.2 ml PBS/mouse). Once the subcutaneous tumor reached 1–1.5 cm in diameter, it was removed and cut into about 1–2 mm^3^ cubes, which were implanted by micro-surgical method into right lung of additional recipient nude mice, after which the animals were randomized to receive either G31P (0.5 mg/kg on alternative days, s.c.) or vehicle (saline solution, s.c.) at one week after implantation (8 per group). Tumor tissues were harvested 5 weeks after tumor implantation for further experiments. Tumor volumes were calculated using the formula: volume = (length × width^2^ /2), while microvessel density (angiogenesis) was calculated as the micro-vessel length per tumor area, determined by ImagePro Plus 6.0 software (Microsoft Media Cybernetics, Bethesda, MD, USA).

### TUNEL staining

Assessment for apoptosis was conducted using a commercially available TUNEL assay kit (Keygen Biotechnology, China). Briefly, sections were deparaffinized, digested with proteinase K (20 μg/ml) at 37°C for 15 minutes, and soaked in PBS for 5 minutes. DNA fragmentation was detected using TUNEL apoptosis detection kit which specifically labeled 3′-hydroxyl termini of DNA strand breaks using red fluorescent protein (RFP)-conjugated dUTP. Each section was covered with labeling solution for 1 h at 37°C in a humidified chamber. RFP labeled DNA fragment were observed with a fluorescence microscope (BX-51, TR32000 Olympus, Japan).

### Immunohistochemistry

Immunohistochemistry was performed as described previously [24]. In short, tissue sections were deparaffinized in xylene and rehydrated with ethanol. Tissue sections were pre-incubated with 10% normal goat serum in PBS (pH 7.5) followed by incubation at 4°C with primary antibodies specific for PECAM-1/CD31 (1:100, Bioworld technology, USA), NFкB-p65 (1:200, Santa Cruz, USA) or VEGF (1:300, Santa Cruz). After PBS washes, samples were incubated with biotin-labeled secondary antibodies for 30 minutes at room temperature, then the target mediators were visualized with peroxidase-labeled streptavidin-complexes/DAB, which gave a yellow-brown stain to positive tissues; the sections were briefly counterstained with hematoxylin, mounted and visualized under an inverted microscope (Leica, Germany). Immunostaining intensity was calculated by using a semi-quantitative method with software Image-Pro Plus 6.0 (Microsoft Media Cybernetics, Bethesda, MD, USA).

### Immunoblottings

Same protocol was used as described previously in our studies using PVDF membranes [25]. Blots were incubated overnight with primary antibody at 4°C. Primary antibody dilution is as follow: CXCR2 (1:1000), AKT (1:1000), pAKT (1:1000), ERK1/2 (1:1000), pERK 1/2 (1:1000), p65 (1:1000), PARP (1:5000), Caspase-8 (1:1000), Bax (1:1000), Bcl-2 (1:1000), and VEGF (1:1000). Secondary antibody was used (HRP conjugated anti-rabbit or anti-mouse antibodies) for 1 h at room temperature. Anti-GAPDH (1:1000) or anti-α-Tubulin (1:1000) was used to confirm equal loading. Immunoreactive proteins were visualized with the ECL detection system (Advansta, USA) on a ChemiDoc MP imaging system (Biorad, USA).

### Statistical analysis

All data are presented as mean value ± Standard error of the mean (SEM). Comparisons to analyze variance among multiple groups were made with one way analysis of variance (ANOVA). Student's *t*-test was used to compare two different groups. Both statistical analyses were performed using GraphPad Prism 5.03 and *p* < 0.05 was considered significant (*).
